# Presence of Micropollutants and Transformation Products During Subsurface Irrigation with Treated Wastewater Assessed by Non-Target Screening Analysis

**DOI:** 10.1021/acsestwater.4c00930

**Published:** 2025-01-15

**Authors:** Alessia Ore, Rick Helmus, Dominique M. Narain-Ford, Ruud P. Bartholomeus, Nora B. Sutton, Annemarie van Wezel

**Affiliations:** † Environmental Technology, Wageningen University & Research, 6708 WG Wageningen, The Netherlands; ‡ Institute for Biodiversity and Ecosystem Dynamics, University of Amsterdam, 1098 XH Amsterdam, The Netherlands; § National Institute for Public Health and the Environment, P.O. Box 1, 3720 BA Bilthoven, The Netherlands; ∥ KWR Water Research Institute, 3430 BB Nieuwegein, The Netherlands; ⊥ Soil Physics and Land Management, Wageningen UR, 6700 HB Wageningen, The Netherlands

**Keywords:** micropollutant transformation products, biodegradation, non-target analysis, subsurface irrigation, groundwater quality, mobile compounds, water reuse

## Abstract

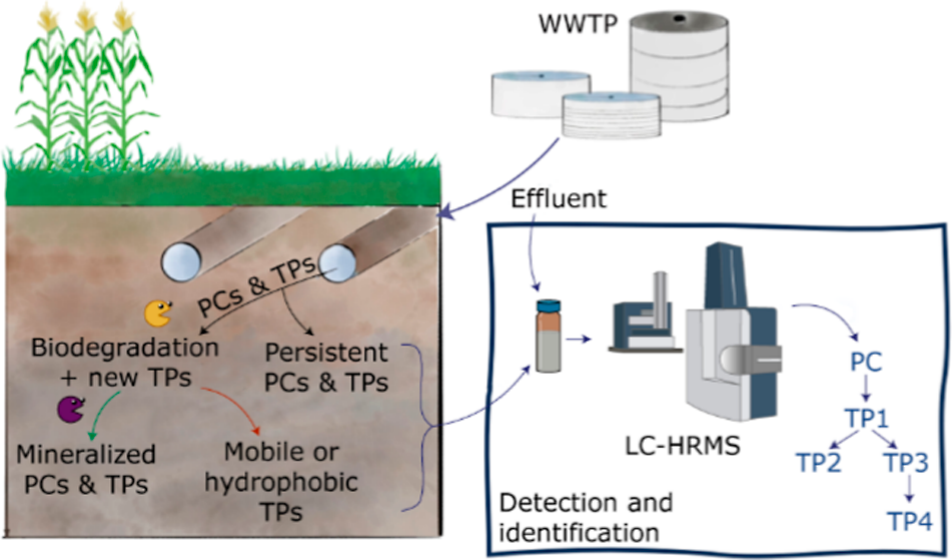

While wastewater treatment plant (WWTP) effluent offers a potential alternative source for irrigation, the fate of organic micropollutants (OMPs), including transformation products (TPs), in effluent-irrigated fields remains largely unknown. Using non-target analysis (NTA), we investigated OMPs in WWTP effluent and their distribution throughout a full-scale subsurface irrigation (SSI) field where effluent was used for irrigation. Our results indicate that TPs accounted for approximately 80% of the detected effluent OMPs. Weather and SSI hydrology seem to influence OMP distribution and transformation. Wetter conditions promoted deeper leaching of OMPs in soil, and drier conditions favored their capillary rise and biotransformation, as shown by the detection of 37% more TPs in the rhizons during a dry year. On average 45 OMPs, at least 50% with a logD <3, were detected at −2.3 m depth, highlighting their potential to reach groundwater and the importance of including TPs in further risk assessment. This approach demonstrates how NTA and subsequent data analysis tools can support the identification of (unknown) OMPs and contribute to understanding OMP fate under field conditions, which is the first step in an exposure-driven environmental risk assessment. Overall, our study emphasizes the importance of carefully considering (unknown) OMPs for more responsible effluent reuse.

## Abstract 2

Through novel methods, we investigated the presence of micropollutants and unknown transformation products in wastewater treatment plant effluent and a field reusing the effluent via subsurface irrigation.

## 1 Introduction

Reuse of alternative water sources is a way to tackle freshwater scarcity, however, it is important to consider water quality to ensure safety. One alternative water source for agriculture is wastewater treatment plant (WWTP) effluent, which could be reused as irrigation water, given its predictable availability and monitorable quality.^1^ The intentional reuse of WWTP effluent can satisfy a portion of the freshwater demand for agriculture, accounting for 70% of the global freshwater withdrawal.^2^

Reusing WWTP effluent poses potential risks to the environment and groundwater due to the presence of potentially hazardous contaminants. Conventional wastewater treatment processes are not fully effective at removing all organic micropollutants (OMPs), even with more advanced physical-chemical technologies,^3,4^ due to the diverse OMP characteristics and the limitations of treatment conditions. A portion of OMPs often remains in the WWTP effluent,^1,5−7^ including pesticides, pharmaceuticals, PFAS, personal care products, and their transformation products (TPs).^8,9^ Notably, some TPs in the effluents may exceed the parent compounds (PCs) concentration and toxicity, highlighting the need for further investigation into their presence.^3,10,11^

The EU regulation for water reuse^12^ encourages the use of treated wastewater in irrigation and addresses the minimum requirements for reuse. While OMPs, like pesticides and pharmaceuticals, are mentioned as additional requirements to manage human and environmental risks, no specific compounds or concentrations are referenced. European water guidelines^13,14^ and recent revisions^15,16^ include some contaminants, but the lists are not representative of the variety of compounds in the effluent and overlook the majority of TPs.^9,17^ TPs are often sparingly included as many remain unknown or are difficult to detect with the methods conventionally applied due to low concentrations and the lack of analytical standards.^18,19^ Consequently, many OMPs go unnoticed in effluent discharge or reuse. Although it is not feasible to include all the relevant TPs and OMPs in water treatment regulations, more comprehensive water quality monitoring is required to better assess WWTP efficiency and the feasibility of effluent reuse in agriculture.^1^

Subsurface irrigation (SSI) makes use of shallow underground pipe systems at depths of approximately 1 m below ground level, used for field drainage during wet periods. Flow can be reversed during dry periods, allowing infiltration of water, or in this case effluent.^20,21^ Capillary action toward the plant roots and evapotranspiration by the plants result in the use of irrigated water within the field.^20^ During SSI, effluent OMPs come in contact with soil and groundwater, where they may undergo biotic (i.e., biodegradation) or abiotic (e.g., hydrolysis) transformation, generating TPs or fully converting into inorganic substances.^18^ Therefore, it is important to understand OMPs’ fate following irrigation via SSI. Soil passage via SSI could act as additional treatment improving effluent quality via (bio)degradation,^22,23^ but generally more mobile TPs are produced, potentially including persistent and toxic compounds.^10,11,24^ Biodegradation plays a key role in OMP fate in natural systems, but its efficiency depends on several factors, like microbial community composition, organic carbon availability, redox condition, and retention time.^18,25,26^ Moreover, some released OMPs may resist biodegradation and persist in the environment. Mobile and persistent contaminants, including TPs, could reach groundwater and compromise its quality.^27,28^

To understand if effluent reuse with SSI is a viable option for water reuse, we investigated the OMPs in a WWTP effluent used in an SSI agricultural field, aiming to clarify its composition. Furthermore, we studied the presence of released OMPs and related TPs, coming from the effluent or formed in the field, in groundwater samples from the SSI field. In this study, raw non-target analysis (NTA) data were analyzed to detect OMPs in both WWTP effluent and groundwater samples. NTA allows tentative identification of unknown compounds, like TPs. Comprehensive TP screening workflows with patRoon 2.3^29^ were used to facilitate the data treatment, including automated TP screening. The work builds upon and complements previous research in the same agricultural field, in which OMPs were quantitatively studied using target analysis.^22^ This study addresses the knowledge gap on (unknown) contaminants in the effluent that could potentially pollute soil and groundwater. More information on the effluent composition and OMP fate in irrigated fields can support a more proper prioritization of compounds to be removed from effluents and the selection of suitable indicators to assess WWTP efficiency and effluent suitability for reuse.

## 2 Methods

The analyzed NTA data come from an SSI field in Haaksbergen, The Netherlands, and the adjacent municipal WWTP (Supporting Information SI-I). The WWTP applies standard secondary treatment technologies (suspended solids removal and biological treatment) to the water received from a combined sewer system. The total average residence time of water in the WWTP is approximately 20 h during dry weather conditions and 3.5 h during heavy rain. The WWTP effluent was reused from 2015 until 2022 during the growing season of feed crops via SSI, without residence in intermediate buffer basins. The SSI system consisted of parallel pipes located at −1.2 m depth and 6 m apart from each other (Supporting Information SI-I). The data set included NTA data from two monitoring points in the SSI-irrigated field, each sampled at four depths in 2017 and 2019 when corn was grown on the field. The selection of the sampling location, depths, and years analyzed is based on the results of a chloride/bromide ratio test^30^ to identify the locations receiving the WWTP effluent and on the previous research by Narain-Ford et al. (2022),^22^ who observed most removal of target OMPs between infiltration pipes. We chose to compare a location close to a pipe and one in between pipes to investigate TP presence as well as effluent OMP transformation and spreading in the field.

### 2.1 Study Area and Samples Collection

The Haaksbergen SSI system and the sampling methods have been previously described by Narain-Ford et al. (2022).^22^ The system has been monitored since 2015 to study its hydrology and follow effluent infiltration and OMPs’ fate using targeted analyses.^22,23,31^ The effluent composition tends to be constant over time in terms of OMP presence, however, OMP concentration is affected by precipitation and temperature over year-round periods. Additionally, these earlier studies highlight the complexity of the hydrological fluxes in the system, which affect OMPs’ fate.

The present research focuses on NTA data analysis from samples collected in 2017 and 2019. Considering the precipitation and reference evaporation data^32^ from January first to the sampling date, the potential precipitation surplus was 3.4 mm in 2017, while 2019 was dryer with a deficit of 23.3 mm. The 24 h composite WWTP effluent and water samples from two locations in the SSI field, taken at depths of −0.6 (i.e., rhizons, unsaturated zones above the level of infiltration), −1.3, −1.8, and −2.3 m, (minifilters in the saturated zone) were selected for the NTA data treatment and interpretation. One replicate per effluent and field sample was available. The locations are hereafter referred to as *Close* and *Between*, referring to their position close-to an SSI infiltration pipe and in-between pipes, similar to Narain-Ford et al. (2022)^22^ (Supporting Information SI-I).

The samples were collected in 250 mL HDPE bottles, stored at −20 °C immediately after reaching the laboratory on the sampling day, and thawed on the day of analysis. Effluent samples were collected on July 3^rd^, 2017, and July 11^th^, 2019, whereas the field samples were collected on July 3^rd^, 2017 (during subirrigation) and October 7^th^, 2019 (a few days after the end of the subirrigation period). Rain episodes were registered during the three days preceding the effluent samplings: 23.5 mm in 2017 and 0.5 mm in 2019.^32^ This selection of samples provided the most comparable NTA data between the two years and the position relative to infiltration pipes.

### 2.2 NTA Samples Preparation and Data Acquisition

The sample preparation for direct injection and the analysis in the LC-HRMS system, which produced the NTA data analyzed in this research, were performed by KWR Water Research Institute and are described in detail in Supporting Information SI-II. The LC-HRMS method has been used and validated in previous research.^19,33^ Briefly, a C18 column was used for liquid chromatography and an Orbitrap with an electrospray ionization source was used for mass spectrometry. In spring 2022, the samples were analyzed in positive and negative ionization modes along with blanks and performance standard (Table S2) samples containing target OMPs at known concentrations.

### 2.3 NTA Data Processing with PatRoon

The NTA data from the selected samples were processed with patRoon 2.3.2, an open-source R-based workflow for non-target data analysis and automated TP screening.^29,34^ HRMS blanks and performance standards were included in the patRoon data set to ensure high-quality results. The workflow included several steps (Section 2.3.1) and simultaneously analyzed positive and negative ionization data. For some of the steps, default values were optimized based on the NTA equipment, following what was previously described in Helmus et al., 2021.^34^

#### 2.3.1 General Description of the Workflow

Two patRoon scripts (available in full in the Supporting Information ST1 and ST2) were used to treat the raw data obtained from the LC-HRMS analysis of the effluent samples (ST1) and the field samples (ST2). A simplified version of the workflow (without TP screening) was used to analyze the NTA data of the performance standard samples, using the same parameters applied to the effluent and field samples (bottom of ST1).

The workflows include the following steps and tools. Data transformation from *.raw* to .*mzML*^35^ format was performed through ProteoWizard,^36^ whereas the feature finding and sample grouping steps were done using OpenMS.^37^ The sample grouping step produces feature groups, consisting of features that are considered equivalent across multiple samples.^34^ Subsequently, filtering steps (e.g., minimum intensity thresholds and blank removal) were applied to clean the data. A suspect screening step was included in the workflow to check for the presence of the OMPs previously detected via target analysis over a 20-month period by Narain-Ford et al. (2022)^22^ in the Haaksbergen SSI field. These OMPs (46 pesticides, 29 pharmaceuticals, and 14 industrial chemicals listed in Supporting Information SI-III) were provided in a suspect list to the software and were considered as PCs for the subsequent TP screening step. Then, we performed a suspect screening looking for TPs of the full list of OMPs previously detected. In the TP screening, BioTransformer^38^ with the microbial degradation module enviPath^39^ was used to generate a list of TPs (up to the second generation—Table SE-1) that may originate from the PCs following biological transformation processes by environmental microbiota. After filtration for TPs of greater interest (see ST1, ST2), this list was used to screen for predicted TPs in the samples. With the filters, we removed second-generation TPs with a mass ratio TP/PC lower than 60%. A componentization step was then included, and unwanted adducts and isotopes were manually removed from the data set. The following annotation step used GenForm^40^ and MetFrag^41^ to assign a formula, a compound candidate, and an identification (ID) level to the peaks in the feature groups. The ID estimation in patRoon is based on Schymanski et al. (2014)^42^ with some modifications on the rules to assign the level, as reported in Supporting Information SI-IV and the patRoon handbook and manuals.^43,44^ Briefly, in a suspect screening, Schymanski et al. assign the TP a starting ID level of 3, whereas in patRoon the starting level is 5. The level can improve in both cases with extra information retrieved from the analysis. Level 1 is only assigned if the suspect is confirmed by an analytical standard. All feature groups with assigned ID level 5 were removed from the data set to improve the reliability of the results. Finally, PCs and TPs were linked to visualize compound transformation pathways in the final report. The feature groups were manually reviewed in patRoon to further clean the data set by removing incorrectly integrated peaks before generating HTML reports.

Additional filters (ST1, ST2) were applied to the feature groups to obtain a simplified data set to generate figures and tables. The filters prioritized duplicate feature groups assigned to the same suspect and multiple suspects assigned to the same feature group. In these cases, only the feature group or suspect with the highest ID level was retained for plotting purposes. The MS(/MS) spectra fragments of the compounds in PC- and the performance standard lists were compared with those from MassBank^45^ and PubChem.^46^ This last step ensured proper identification and allowed the assignation of ID level 1 to the suspect OMPs detected in both the performance standard and effluents and field samples (Tables SE-4, SE-5, and SE-6). A comparison of the TPs detected in the data set with the TPs registered in the PubChem Transformations database^47^ (which includes transformation reactions from literature) was performed, the overlapping TP results are provided in Tables SE-2 and SE-3.

Experimental pH-dependent logKoc values would have been ideal for assessing the mobility of ionizable compounds like the OMPs and TPs discussed in our study. Given the absence of this data for most TPs, logD was used to represent the hydrophobicity-driven portion of sorption,^48,49^ which was the best approximation in our case. The charge was included to consider electrostatic interactions in soil, which is predominantly negatively charged. Charge and logD values at pH 7 (Table SE-7) were plotted against the distance between each sampling point and the infiltration pipe. The logD values of OMPs detected in the field were retrieved using the InChIKey from patRoon reports. Whenever possible, the InChIKey was converted to SMILES via the Chemical Identifier Resolver,^50^ then used to predict the charge and logD of each contaminant with Chemicalize.^51^ For the location close to the pipe, the plotted distance corresponds to the vertical path between the pipe and the sampling point. For the location between pipes, the diagonal distance was used. The rhizons and the sampling points at −1.3 m below ground level share the same distance from the pipe, resulting in overlap when plotted.

### 2.4 Data Interpretation

Excel and R (Tidyverse packages) were used to treat the data obtained from the HTML reports. Additionally, the databases PPDB,^52^ enviPath,^39^ DrugBank,^53^ EU Pesticides Database,^54^ EAWAG-BBD (biocatalysis/biodegradation database),^55^ MassBank^45^ and PubChem^46,47^ were used to manually interpret the PCs and TPs detected and the transformation reactions involved.

The authors recognize some limitations in the present study. The TP prediction tool used in patRoon (Biotransformer) may have overlooked possible TPs, causing the TP-suspect list to be insufficiently inclusive. Alternatively, TPs may have been missed due to too high polarity causing early elution, low concentrations falling below detection limits, or peak intensities below the scripts’ cutoff. TPs outside the chemical space handled by LC-HRMS would also be missed in our analysis. In designing the patRoon scripts we aimed to balance the comprehensive screening of TPs with minimizing the introduction of uncertainties. The filters applied on the list used for TP screening (see previous section) helped prevent the erroneous assignment of small, nonspecific fragments to a specific PC, particularly when such fragments could originate from multiple PCs, but this could have resulted in overlooking of TPs formed later on in the transformation pathway. Target analysis would be needed for further confirmation of the TP results, but is often hampered by the lack of knowledge on TP formation in the field (to which the study intends to contribute) and by the availability of analytical-grade TP standards.

Our data set includes only one replicate per sampling point per year. Although we recognize that this is a small number of samples for a field study, the results are backed up by previous research in the same field and period and employing target analysis. Therefore, this study adds complementary information on TPs to the study of Narain-Ford et al.^22^

Our transformation reaction analysis is based on the predictions of BioTransformer (EAWAG-BBD Pathway Prediction System^55^) and the TPs detectability and is different from canonical approaches studying OMP biodegradation pathways. To identify the types of reactions that produced the TPs detected in the data set, we used the elemental changes in the PC-TPs components and the EAWAG-BBD^55^ pathway prediction rules, both through patRoon and manual verification. In this research, with the term OMPs we refer to contaminants in general, which can be either PCs or TPs.

## 3 Results and Discussion

This study addresses the release of OMPs in WWTP effluent in an SSI-irrigated field through an NTA approach, aiming to shed more light on OMPs in the effluent and the field. In the first part of the Results and Discussion, the OMPs detected in the effluent are analyzed. In the second part, we focus on the OMPs detected in the SSI field, and in the third part, we investigate the transformation reactions behind the detected TPs.

### 3.1 Variability in the Presence of Parent Compounds and Transformation Products in the WWTP Effluent

**Figure 1:**
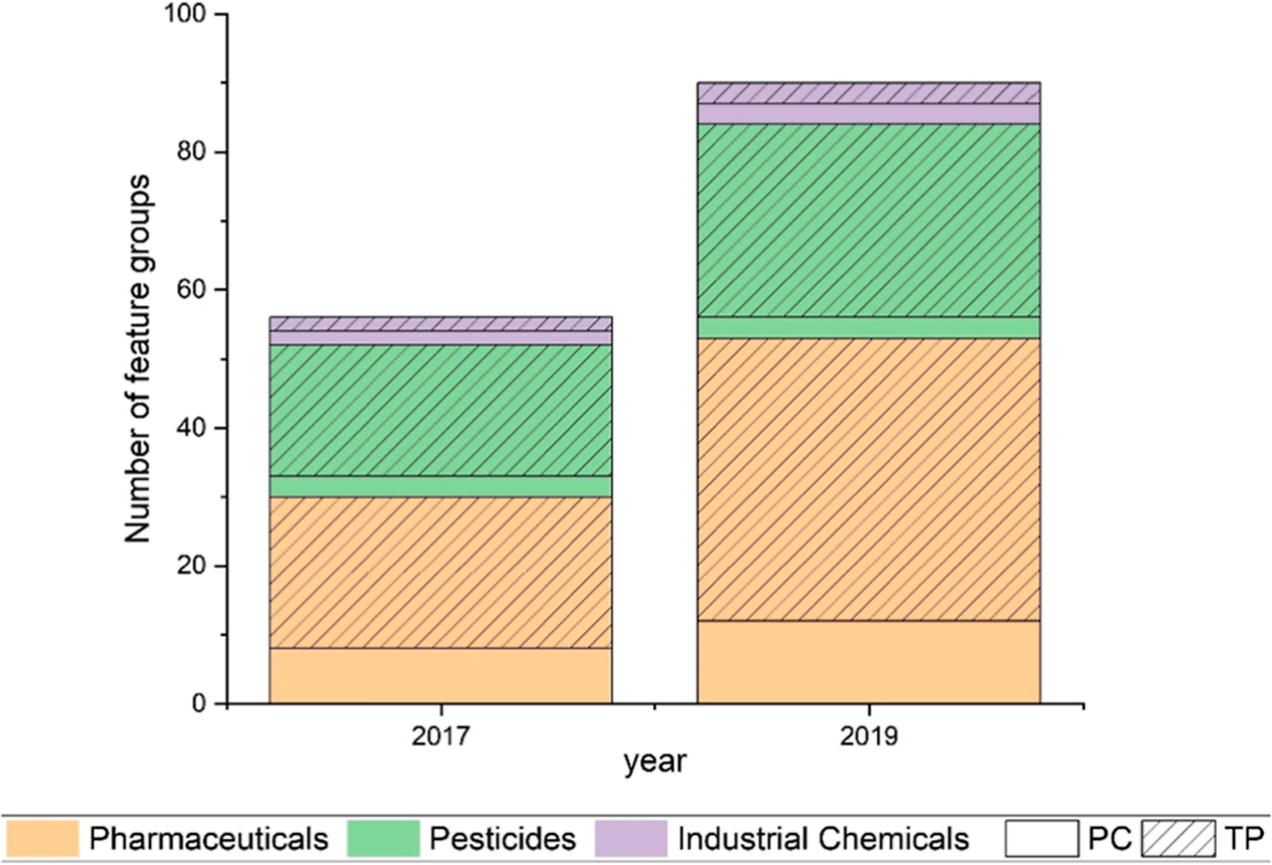
Feature groups corresponding to OMPs detected in the WWTP effluent in the normal precipitation (2017) and dry (2019) years, differentiated per contaminant type and in PCs and TPs.

A high proportion of the feature groups detected in the WWTP effluent samples was annotated as TPs, which are often overlooked in water quality monitoring programs. In 2017, feature groups corresponding to a total of 56 OMPs were detected, 43 of which were recognized as TPs; in 2019, 90 compounds, including 72 TPs, were found in the effluent (Figure 1, for more information on the OMPs, including ID level estimation, see Table SE-4). Eighteen of the 78 suspect TPs detected in the effluent data set (including both years) are registered in the PubChem transformations database^47^ (Table SE-2). Our results show that to fully assess effluent quality, monitoring should not only consider PCs but also a variety of TPs. We detected mostly pharmaceutical and pesticide TPs in the effluent samples of both 2017 and 2019, including pharmaceutical TPs that were not human metabolites and likely formed during wastewater treatment processes (Table SE-4). The PCs detected in the effluent samples are likely resistant, at least to a certain extent, to the treatment technologies applied. Examples, detected in our data set and confirmed by literature, are diclofenac,^56^ furosemide,^57^ 1H-benzotriazole,^6^ and acesulfame.^58^

For water reuse purposes, it is important to shed more light on TPs in the effluent. Most available studies in literature have focused on TPs from specific compounds and did not provide information on the wide range of TPs actually present in WWTP effluents. For instance, Letzel et al. (2015)^59^ studied the presence of sartans (blood pressure regulators) and their TPs in WWTP effluents, while Lei et al. (2021)^60^ surveyed the benzodiazepines (anxiolytic drugs) and the TPs generated in 11 WWTPs. In our data set, other blood pressure regulators (e.g., metoprolol) and mood-control (e.g., venlafaxine) drugs were detected, but no sartans or benzodiazepines specifically. While these studies provide insights into TP presence, they do not comprehensively assess effluent quality. In contrast, Beretsou et al. (2022)^61^ conducted a more extensive study of OMPs in the influent and effluent of WWTPs, reporting 55 target compounds in the effluent, 15 of which were TPs, mainly deriving from pharmaceuticals. By analyzing the influent composition, they demonstrated that 8 of these TPs in the effluent were formed during the WWTP processes. Among these, tramadol-N-oxide, carbamazepine TPs, and venlafaxine TPs were also detected in our data set. Although our study did not focus on the formation of TPs within WWTPs, Beretsou et al.^61^’s findings support our observation of newly formed pharmaceutical TPs in the WWTP effluent.

In our study, precipitation seemed to affect effluent quality in WWTPs, potentially diluting some OMPs to concentrations below the detection limits and/or reducing WWTP transformation efficiency due to shorter residence times.^4^ In 2017, 23.5 mm of rain was recorded over two days before sampling, compared to just 0.5 mm in 2019.^32^ This likely contributed to the detection of 34 more feature groups (5 annotated as PCs and 29 as TPs) in 2019 than in 2017, with almost twice as many pharmaceutical TPs and one-third more pesticide TPs detected in 2019. For reuse purposes, it is advisible to perform effluent characterization with NTA during dry weather conditions (when more OMPs can be detected) as a realistic worst-case estimate of its impact on the receiving environment.

### 3.2 Presence of Contaminants in the Field

As a result of reuse in SSI systems, effluent contaminants enter soil and groundwater. In the subsurface, OMPs can undergo biological or abiotic transformations under different conditions compared to those in WWTPs, potentially resulting in the formation of different TPs. Thus, we performed a comprehensive non-target analysis of OMPs in the field irrigated with effluent via SSI. We present the OMPs detected in the SSI field, along with their mobility and the transformation reactions likely involved in TP formation, either in the field or the WWTP. More information on the compounds is present in SE, including the tentative chemical formula and structure of the OMPs, their estimated ID level, and the peak intensities per sample (Table SE-5).

#### 3.2.1 OMPs in the Field

**Figure 2:**
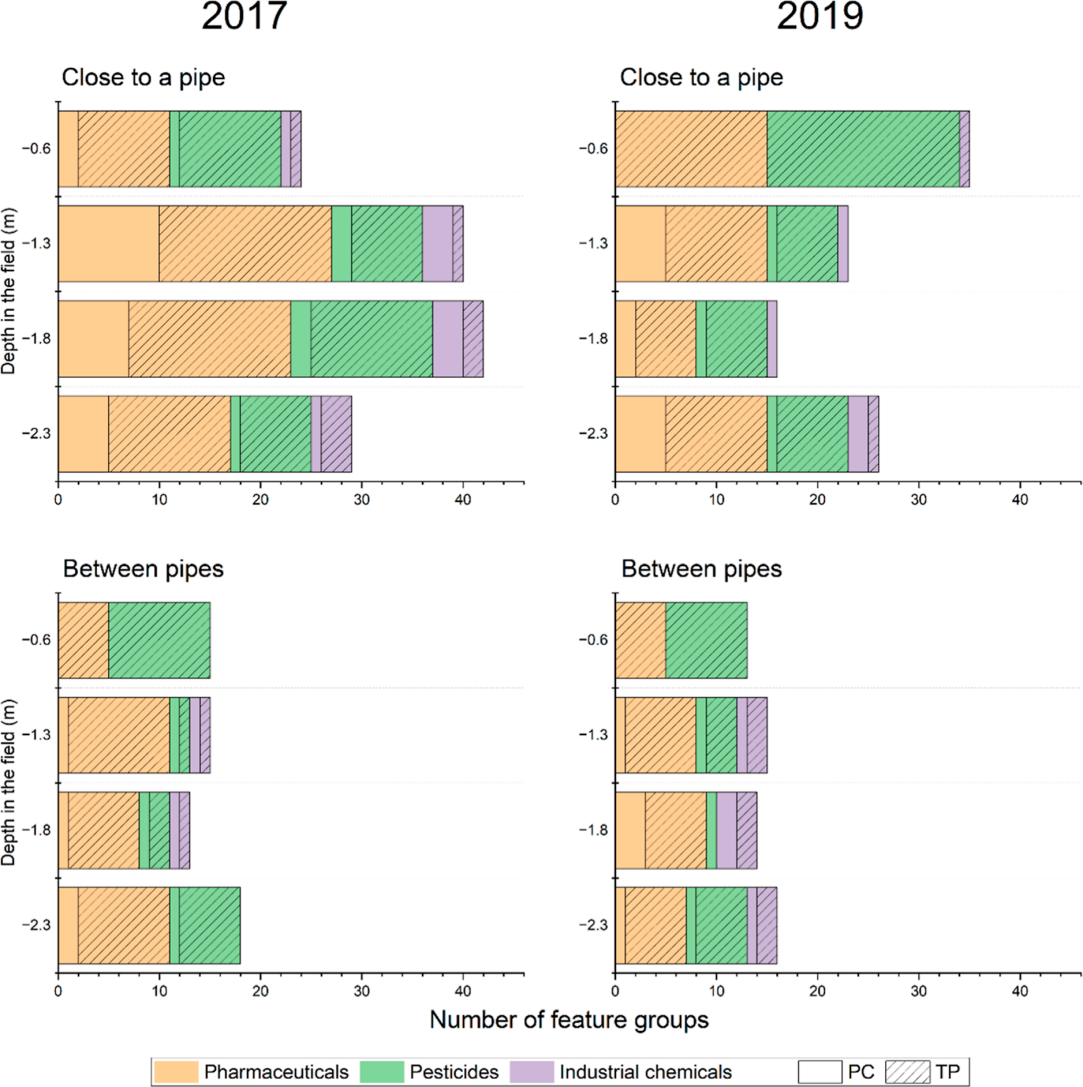
Distribution of OMPs in the SSI field in 2017 and 2019.

Our results indicate that weather conditions and soil hydrology likely influence OMP distribution and TP formation in the SSI field. A total of 121 feature groups assigned to OMPs were detected in the field, with similar numbers in 2017 and 2019 (92 and 80, respectively, Table SE-5). In general, TPs represent the majority of OMPs at all depths in the whole data set, but OMP distribution in the field varies based on the location. A higher number of OMPs (118 on average between the two years) and a greater proportion of PCs (24%) were detected close to the infiltration pipes, whereas in between pipes, fewer OMPs were detected (60 on average, with 18% of PCs), more or less evenly distributed over the sampled depth in both years (Figure 2). At the rhizons between pipes, only TPs were detected.

Close to the infiltration pipe, we observed different trends between the two years which may be traced back to the weather effects on SSI hydrology. In 2017, more OMPs were detected between −1.3 and −1.8 m. The field samples of 2017 were taken after a heavy rainfall^32^ that probably caused the leaching of OMPs to these depths. The hydrology of the system is complex,^23^ but in more wet conditions, the lateral water fluxes to the stream and downward fluxes increase, whereas upward movement toward the rhizons decreases.

In 2019, most OMPs were detected in the rhizons. In this year, the groundwater table was considerably lower (on average 24 cm lower than in 2017)^62^ and might have favored the capillary rise of infiltrated water with OMPs.^23^ A higher TP formation is also likely due to higher retention times and more aerobic conditions in the SSI system. In dryer conditions, besides an increased capillary rise, there is a slower movement of water laterally and downward. The capillary rise leads OMPs to more oxygenated zones where biodegradation is faster and more efficient,^25^ and slower water movement leads to higher retention times and thus possibly more biodegradation. Altogether, these water movements might explain the higher number of TPs observed in the rhizons in 2019.

Moreover, the results for 2019 can be related to the infiltration of more concentrated effluent during dry periods, which occurred more frequently in 2018 and 2019,^32^ and higher OMP concentrations which may lead to improved biotransformation and TP production.^18^ This conclusion is supported by the previous study of Narain-Ford et al. (2022),^22^ who observed higher background OMP concentrations in the field before the start of SSI in 2019 due to the very dry year of 2018, in which effluent was hardly diluted by rain during the infiltration period. This caused OMPs to reach high concentrations in the soil which could not be completely washed out by precipitations in winter 2018–2019 and spring 2019.

Overall, several OMPs (between 16 and 29) were also detected at −2.3 m in the field (Figure 2). As infiltration occurred at −1.2 m, the detection of OMPs at −2.3 m indicates their transport toward groundwater, probably due to their high mobility and/or persistence.^24^ OMPs detected at the deepest sampling points include highly persistent and mobile PCs and related TPs, for example carbamazepine, venlafaxine, and venlafaxine-TPs, and highly persistent PCs and related TPs (1H-benzotriazole, DEET, and metolachlor-TPs), previously reported to resist removal processes.^22,57^ The deeper groundwater in Haaksbergen is protected by an impermeable loamy clay layer below our sampling depths and by the high lateral flow toward the nearby surface water.^21^ However, these geohydrological characteristics are specific to this site and might not occur in other SSI fields. Therefore, reusing WWTP effluent through SSI requires careful consideration of persistent and mobile OMPs, including TPs, possibly present in the effluent or forming in the soil. Implementing additional measures to reduce the presence of OMPs in the WWTP influent (with responsible chemicals use in households, commercial activities, and agriculture) and effluent (by upgrading WWTPs with advanced OMP treatment technologies) is needed to protect freshwater sources from OMP contamination.^1,63^

#### 3.2.2 Mobility of OMPs in the Field

The charge state and logD values at pH 7 were utilized to infer the mobility of OMPs in the field (Table SE-7). It has to be noted that these data could only be retrieved for a limited subset of the OMPs detected, namely 100% of the PCs and 30% of the TPs.

**Figure 3:**
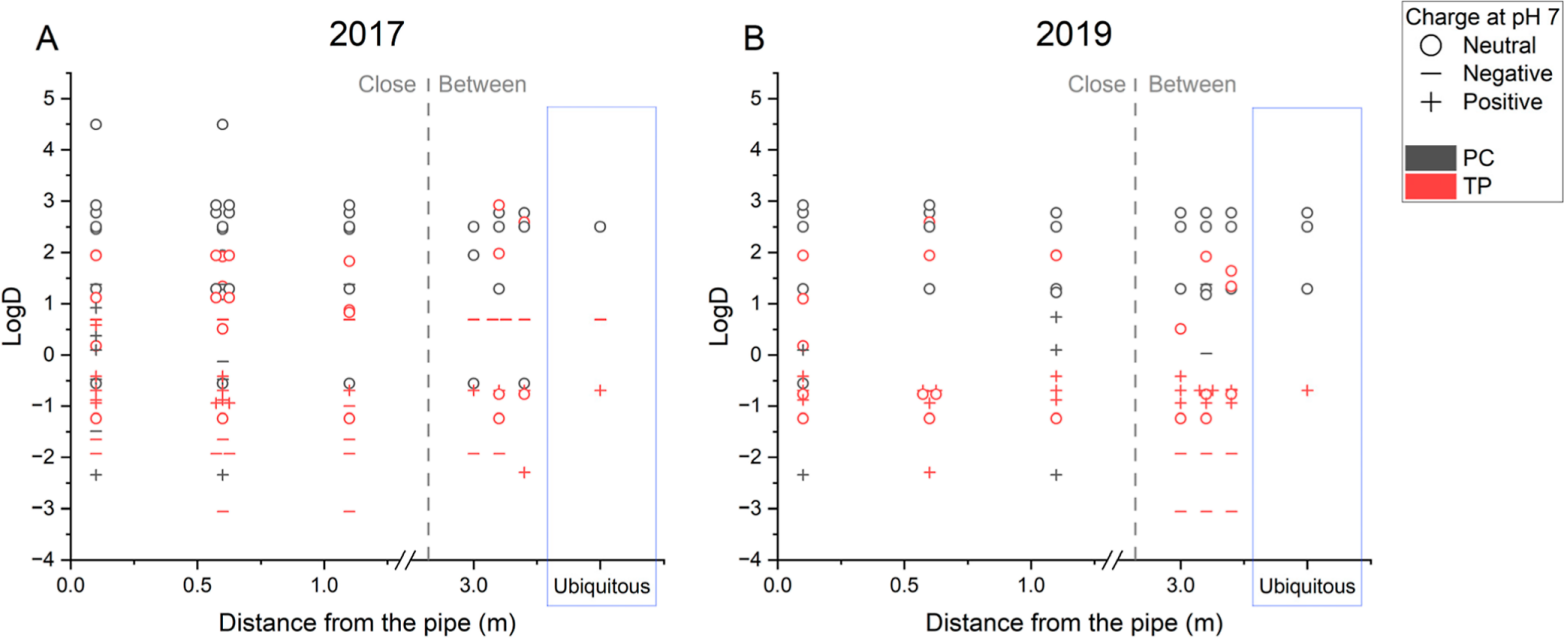
Mobility indicators (logD and charge, both at pH 7) of the PCs and TPs detected in the SSI field in 2017 (A) and 2019 (B) plotted against the distance between the infiltration pipe and the sampling point. The OMPs in the blue box were ubiquitous, i.e. detected at all sampled distances from the pipe.

We investigated the correlation between OMPs’ mobility indicators and OMPs’ detection in the field in Figure 3. Most of the compounds detected with retrievable mobility indicators had a logD at pH 7 below 3, which indicates already moderate mobility.^49^ High logD values (>3) generally indicate low water mobility and a tendency for soil sorption. In SSI systems, OMPs with high sorption affinity remain close to the infiltration pipe and require attention, as they may accumulate in the field.^31^ Typically, in temperate climates, the winter period between growing seasons allows for the restoration of an SSI field to low OMP background levels, aided by winter precipitation and drainage.^64^ However, OMPs with high logD tend to sorb to soil particles, which can result in their retention in the field.

Narain-Ford et al. (2022)^22^ observed an accumulation of highly persistent and low-mobility OMPs near SSI infiltration pipes. Similarly, in our data set, the OMPs with higher logD were detected mostly close to the infiltration pipe (Figure 3). This was more evident in 2017 (Figure 3A), when a greater number of compounds with logD >1 were detected at distances less than 1 m from the infiltration pipe, compared to those at greater distances. Compounds like fipronil (logD = 4.49) and dimethenamid-p (logD = 2.92) were only detected close to the infiltration pipe in both years. Similarly to hydrophobic compounds, cationic OMPs stayed closer to the infiltration pipe, likely due to higher affinity for the negatively charged components of the soil matrix. This behavior is clear for PCs like sotalol (logD = −2.34) and tramadol (logD = 0.1), which, despite their low logD, were detected only in the well close to the infiltration pipe (Figure 3).

OMPs with lower logD values, and higher mobility, are of concern due to their potential to reach deeper groundwater aquifers. Very mobile compounds (logD <0), most of which TPs, were well distributed over the sampling points in the field. In 2019, very mobile TPs were predominant in the well between two pipes, some of which were not detected closer to the infiltration pipe, like triethyl-phosphate-TP2 (diethyl phosphate) and TP5 (ethyl dihydrogen phosphate), with logD −1.93 and −3.05, respectively. Mobile compounds can travel considerable distances along with the water flow. Water movement in SSI is a combination of the fluxes of irrigation, drainage, natural groundwater flow, and capillary rise, causing water (and solutes) to move in different directions. Each of these fluxes is influenced by different conditions, among which are recent weather and SSI operations. This suggests that the OMP distribution in the field depends not only on mobility but also on other factors. For example, in dry periods, the capillary rise of water is higher than in wet conditions.^23^ Recent effluent infiltration leads to more OMPs (of all mobility classes) being detected close to the pipes. Other factors are dispersion^65^ and in situ transformation processes,^19^ which might form new TPs with different mobility than the PC at any distance from the infiltration pipes.

Figure 3 clearly shows how TPs are more mobile than PCs, with PCs prevalent in the top part of the plots, and TPs with lower logD values. The identification and monitoring of more TPs and further evaluation of their persistency and possible toxicity are crucial for the safeguarding of deeper groundwater reservoirs as their high mobility may allow them to travel considerable distances.

### 3.3 Transformation of OMPs

Feature groups identified as TPs from many OMPs previously found in the SSI field were detected in our data set (Table 1). Twenty-three out of the 101 TPs detected in the field data set are registered in the PubChem transformation database^47^ (for more information on the feature groups corresponding to these TPs, including their ID level estimation, see Table SE-3). The number of TPs detected per OMP class is similar in the two years, except for the pharmaceutical class, where a greater number of TPs was detected in 2017. Only a portion of TPs detected in the field were also found in effluent samples, suggesting that some transformation may occur in the field. However, considering the limited number of effluent samples analyzed, the specific location of TP formation remains uncertain. Nonetheless, studies report OMP removal in the environment via different mechanisms, with biodegradation as the primary process, alongside sorption, abiotic transformation, and dilution.^4,18,25,66−68^ Therefore, it is reasonable to assume that OMPs released by the WWTP effluent undergo (further) transformation in the SSI field.

**Table 1:** List of OMPs for Which TPs Were Detected in the Field in 2017 and 2019

pharmaceuticals	# TPs 2017	# TPs 2019	pesticides	# TPs 2017	# TPs 2019	industrial chemicals	# TPs 2017	# TPs 2019
1-hydroxyibuprofen	2	1	2,6-dichlorobenzamide	1	2	HFPO–DA	0	1
2-(methylamino)pyridine	2	1	4,6-dinitro-o-cresol	1	1	*N*-phenyl urea	1	1
3-hydroxycarbamazepine	2	2	ametryn	1	1	triethyl phosphate	2	2
atenolol	4	4	atrazine	1	0			
bezafibrate	2	1	cyanazine	2	2			
caffeine	1	1	DEET	2	1			
climbazole	1	0	desmetryne	1	1			
clindamycin	4	3	dimethametryn	1	1			
clofibric acid	1	2	diuron	1	1			
diclofenac	1	0	ethofumesate	0	4			
dimethanamid-p	1	0	fenuron	2	1			
gemfibrozil	0	1	linuron	1	1			
ibuprofen	3	1	MCPB	1	1			
lincomycin	1	1	MCPP	1	0			
metoprolol	7	8	metolachlor-s	8	8			
paracetamol	1	0	metribuzin	1	0			
sotalol	3	3	nicosulfuron	2	2			
sulfamethazine	1	1	prometon	0	1			
tramadol	3	2	propoxur	1	0			
venlafaxine	3	1	terbacil	1	0			
			terbumeton	1	1			
			triazophos	0	2			

Based on the predictions of the BioTransformer^38^ interface in patRoon, we estimated which type of degradation reactions produced the TPs detected in the data set (Table 2). The transformation pathway prediction is limited to the PCs and TPs linked in the TP componentization step of the patRoon workflow and involves the use of the EAWAG-BBD Pathway Prediction System.^55^ Most of the TPs detected in our study were likely formed via demethylation or oxidation reactions. Oxidation is a very common reaction, often involved in OMP environmental transformation pathways,^24^ especially under aerobic conditions. Demethylation can occur under both aerobic and anaerobic conditions, which might explain why we detected a higher number of demethylated TPs.

**Table 2:** Number of Transformation Products per Reaction Type Identified in the Full Study, in the Effluent or in the Field

	# TPs detected in full study		# TPs detected in effluent		# TPs detected in field	
transformation reaction	2017	2019	2017	2019	2017	2019
cleavage	8	5	1	1	7	4
decarboxylation	1	1	0	0	1	1
dehalogenation	2	2	1	1	1	1
demethylation	11	15	5	9	6	6
hydrogenation	2	2	1	2	1	0
hydrolysis	5	6	1	3	4	3
hydroxyl group migration	2	2	0	0	2	2
hydroxylation	2	2	1	2	1	0
oxidation	8	8	3	4	5	4

It is often reported in literature that aerobic reactions are more favorable for micropollutants biodegradation, but the specific reactions involved in degradation under field conditions are scarcely reported. Our results confirm the prevalence of aerobic biotransformation pathways in a real field setting and present other likely OMP transformation reactions that can occur once WWTP effluent is released in the SSI field.

It has to be noted that the TPs identified may subsequently have undergone further transformation in the field, and we are addressing only first- and second-generation TPs. Therefore, more transformation reactions than those reported here most likely occurred in the WWTP and the field. Nonetheless, our study assesses OMP transformation under real field conditions using TP predictions as included in the patRoon workflow. This approach offers a novel way to unravel in situ OMP transformation pathways and screen for TPs in water reuse scenarios. As TP monitoring in the environment is challenging but important to consider for water reuse purposes, the use of models and prediction tools can help prioritize and identify TPs that pose the greatest risks.^3,10^

## 4 Conclusions

This study provides insights for water reuse by highlighting OMPs’ presence in WWTP effluents and in the field when effluent is applied through SSI. Our results show that approximately 80% of the OMPs detected in the effluent are TPs (Figure 1 and Table SE-4). A thorough understanding of TPs’ presence in effluent and further fate in the environment is required to guide mitigation strategies, such as the responsible use of chemicals and the implementation of advanced OMP removal. Our comparison of effluent quality after heavy rainfall (2017) with dry weather (2019) underlines the importance of conducting NTA effluent characterization in dry conditions. This approach enables a more accurate assessment of effluent reuse feasibility by using a worst-case water quality scenario when OMPs (particularly from households) are not diluted by rainwater.

Our findings indicate that precipitation and soil hydrology likely influence OMP distribution and transformation in the SSI field. Wetter conditions in 2017 promoted downward leaching, while drier conditions in 2019 likely favored capillary rise and enhanced biotransformation due to longer retention times and more aerobic conditions (Figure 2). Our analysis of transformation reactions, using the prediction tools included in our patRoon workflows, confirmed the prevalence of aerobic processes in TP formation and identified other transformation reactions possibly occurring in the SSI field. Demethylation, a reaction possible under both aerobic and anaerobic conditions, was involved in the formation of most first- and second-generation TPs detected in our study.

Mobility (here expressed by the logD) plays a relevant role in OMP distribution, as highly mobile OMPs can move along with the water in the complex SSI hydrological system and potentially reach any distance from the infiltration pipes. We mainly detected highly mobile TPs at the greatest distance from the infiltration pipe, highlighting the importance of considering TPs in OMP risk assessment. Cationic and relatively low-mobility OMPs (e.g., sotalol and tramadol), as expected, remained close to the infiltration pipes and might accumulate in the field.^22^

Future studies building on our approach should consider some improvements for a more comprehensive assessment of OMPs in WWTP effluents and SSI fields. Increasing the number of samples would strengthen the understanding of OMP fate. Additionally, further evaluating effects and exposure, for instance with (eco)toxicity tests, and (semi)quantification of relevant OMPs—prioritized for persistence, mobility, and potential toxicity—would improve the completeness of risk assessment for OMP release via SSI. More investigation on PFAS with dedicated NTA workflows is also advisable to address current concerns about their environmental presence.

Overall, our study highlighted the need for careful consideration of OMP presence and fate in SSI systems and similar applications, supporting decision-making on effluent reuse practices and promoting more conscious future water reuse.

## Supporting Information Available

ST1 patRoon script for effluents data analysis (TXT)
ST2 patRoon script for field samples data analysis (TXT)
SI location picture; LC-HRMS method; suspect list for parent compounds; patRoon ID levels rules (PDF)
SE Excel sheets including the suspect list for TP screening, the suspect TPs registered in the PubChem Transformation database, feature groups detected in the effluents, field and performance standard samples (with ID level and further information), and the mobility indicators of the OMPs detected in the field. NTA_SRT: NTA study reporting tool^69^ for Review Only (XLSX)
