# Supplementary material for: Presence of Micropollutants and Transformation Products During Subsurface Irrigation with Treated Wastewater Assessed by Non-Target Screening Analysis
Source: ACS ES T Water. 2025 Jan 15;5(2):891–901. doi: 10.1021/acsestwater.4c00930 (PMC11833869; doi:10.1021/acsestwater.4c00930)
Supplement: Supplementary file 3 — ew4c00930_si_003.pdf [file ew4c00930_si_003.pdf]

Presence of micropollutants and transformation products during subsurface irrigation with treated wastewater assessed by non-target screening analysis

Alessia Ore [a], Rick Helmus [b], Dominique M. Narain-Ford [c], Ruud P. Bartholomeus [d,e], Nora B. Sutton [a*], Annemarie van Wezel [b]

[a] *Environmental Technology, Wageningen University & Research, 6708 WG Wageningen, the Netherlands*

[b] *Institute for Biodiversity and Ecosystem Dynamics, University of Amsterdam, 1098 XH Amsterdam, the Netherlands*

[c] *National Institute for Public Health and the Environment, PO Box 1, 3720 BA Bilthoven, the Netherlands*

[d] *KWR Water Research Institute, 3430 BB Nieuwegein, the Netherlands*

[e] *Soil Physics and Land Management, Wageningen UR, 6700 HB Wageningen, the Netherlands*

*Email: nora.sutton@wur.nl

I. Sampling location

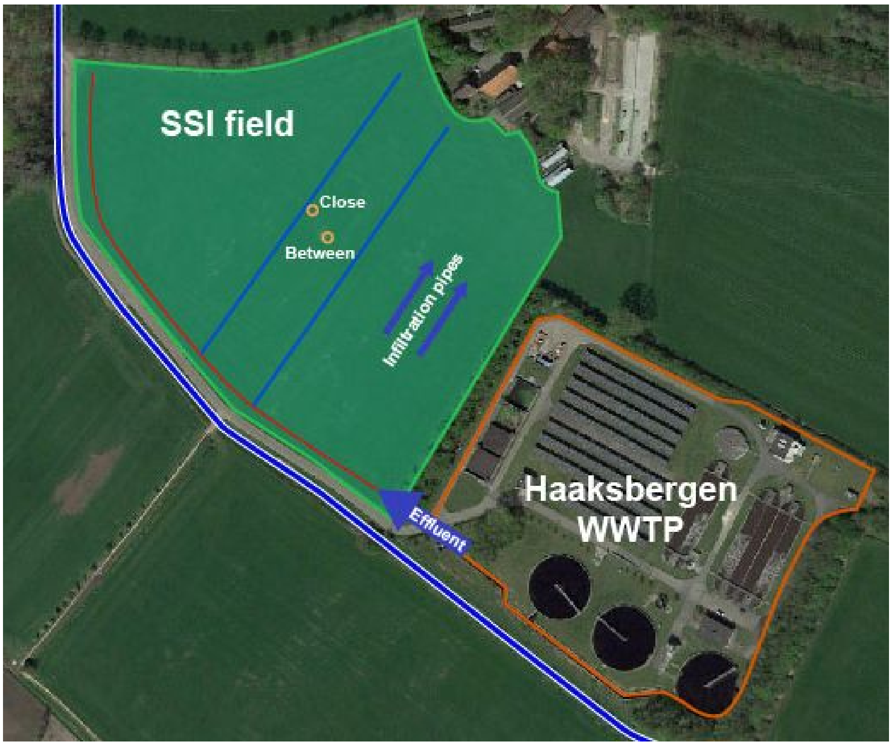

Figure 1. Subsurface irrigated (SSI) agricultural field adjacent to the Haaksbergen wastewater treatment plant (WWTP), the Netherlands. WWTP effluent is provided underground through parallel infiltration pipes (only two are schematically represented with blue lines). The red line represents the pipe connecting all infiltration/drainage pipes. The distance between pipes is not in scale compared to reality. "Close" and "Between" are the analyzed monitoring wells.

II. Samples preparation and LC-HRMS analysis method

The samples were filtered using 0.20 µm Phenex filters (Phenomenex, Torrance, USA) and were spiked with an internal standard stock solution at a final concentration of 1 µg/L before

analysis (Table S1). The LC-HRMS method has been used and validated in previous NTA studies[2,3]. Chromatographic separation of the samples (1x) was performed using an XBridge BEH C18 XP column (150 × 2.1 mm I.D., particle size 2.5 µm, Waters, Etten-Leur, The Netherlands) in combination with a 2.0 × 2.1 mm I.D. Phenomenex SecurityGuard Ultra column (Phenomenex, Torrance, USA). The column temperature was maintained at 25 °C. Eluent A consisted of ultrapure water and 0.05% formic acid. Eluent B consisted of acetonitrile and 0.05% formic acid. The gradient started with 5% B and was held constant for 1 min. Next, the gradient increased from 5% B to 100% B in 24 minutes, and the level of B remained at 100% for 4 min. Then, the level of B was decreased to 5% in 0.5 min, and the column was equilibrated at 5% B for 4.5 min. The flow rate was 0.25 mL/min, and 100 µL of the sample was injected into the LC column. Performance standard samples with spiked target micropollutants (Table S2) and blank samples containing internal standards in ultrapure water (Table S1) were run every 15−20 samples to check for contamination and carryover. A Tribrid Orbitrap Fusion mass spectrometer (Thermo- Fisher Scientific, Bremen, Germany) provided with an electrospray ionization source was interfaced with a Vanquish HPLC system (ThermoFisher Scientific). With every batch run, mass calibration was performed using a Pierce FlexMix calibration solution to obtain a mass error of <2 ppm. The vaporizer and capillary temperature were maintained both at 300 °C, respectively. The RF lens was set to 50%. Sheath, auxiliary, and sweep gases were set to arbitrary units of 40, 10, and 5, respectively. The source voltage was set to 3.0 kV in the positive mode and −2.5 kV in the negative mode. Full-scan high-resolution mass spectra were recorded from 80 to 1300 m/z, with a resolution of 120000 FWHM. The data-dependent acquisition was performed using a high-collision dissociation (HCD) with stepped collision energies of 20, 35, and 50% and an FT resolution of 15000 FWHM.

Table S1. Internal standard list with chemical formula, monoisotopic mass, MS ionization mode, and retention time (RT in the LC column) in minutes, respectively, following the columns from left to right.

**Internal standard**	**Formula**	**M+H/M-H**	**MS ionization**	**RT (min)**
Atrazine-d5	C8D5H9ClN5	221.13243	+	14.47
Atenolol-d7	C14D7H15N2O3	274.21426	+	3.79
Bezafibrate-D6	C19D6H14ClNO4	368.15302	+	15.9
Climbazole-d4	C15D4H13ClN2O2	297.13024	+	14.44
Diglyme-D14	C6D14O3	149.18945	+	5.41
Diuron-d6	C9D6H4Cl2N2O	239.06196	+	14.99
Fenuron-d5	C9D5H7N2O	170.13362	+	9.41
Linuron-d6	C9D6H4Cl2N2O2	255.05687	+	17.17
Metoprolol-d7	C15D7H18NO3	275.23466	+	9.48
Nicosulfuron 3-d6	C15D6H12N6O6S	417.14579	+	12.21
Prometryne-d7	C10D7H12N5S	249.18733	+	14.52
Propazine-d7	C9D7H9ClN5	237.16064	+	16.29
Sotalol-d7	C12D7H13N2O3S	280.17068	+	4.65
Sulfamethoxazole-13C6	C4[13]C6H11N3O3S	260.07952	+	10.77
Tramadol-13C,D3	C15[13]CD3H22NO2	268.21799	+	9.51
Trimethoprim-D9	C14D9H9N4O3	300.20166	+	7.79
Venlafaxine-d6	C17D6H21NO2	284.24912	+	11.06
4-Nitrophenol-2,3,5,6-d4	C6D4HNO3	142.04477	-	11.66
Bentazon-d7	C10D7H5N2O3S	246.09352	-	14.42
Fipronil-(pyrazole-13C3, cyano-13C)	C8[13]C4H4Cl2F6N4OS	438.94485	-	19.92
Fluroxypyr-d2	C7D2H3Cl2FN2O3	254.97140	-	12.84
Furosemide-d5	C12D5H6ClN2O5S	334.03183	-	13.38
Gemfibrozil-D6	C15D6H16O3	255.18728	-	20.09
Ibuprofen-d3	C13D3H15O2	208.14223	-	18.76
MCPA-D3	C9D3H6ClO3	202.03558	-	15.29
Methoxyfenozide-d3	C22D3H25N2O3	370.22155	-	18.36
Pentachlorophenol-13C6	[13]C6HCl5O	268.85986	-	20.73

Table S2. Name, SMILES, and retention time (RT) in the LC column in minutes of the target OMPs spiked in the performance standard samples.

**Name**	**SMILES**	**RT (min)**
1H-benzotriazole	C1=CC2=NNN=C2C=C1	7.96
2,4-Dichlorophenoxyacetic acid	C1=CC(=C(C=C1Cl)Cl)OCC(=O)O	15.26
2,6-Dichlorobenzamide/ BAM	C1=CC(=C(C(=C1)Cl)C(=O)N)Cl	8.18
2-Hydroxyquinoline	C1=CC=C2C(=C1)C=CC(=O)N2	9.16
3-Hydroxycarbamazepine	C1=CC=C2C(=C1)C=CC3=C(N2C(=O)N)C=C(C=C3)O	11.64
4,6-Dinitro-o-cresol	CC1=CC(=CC(=C1O)[N+](=O)[O-])[N+](=O)[O-]	15.91
4-Hydroxyquinoline	C1=CC=C2C(=C1)C(=O)C=CN2	5.98
Acesulfame	CC1=CC(=O)[N-]S(=O)(=O)O1.[K+]	3.19
Ametryn	CCNC1=NC(=NC(=N1)SC)NC(C)C	12.82
Antipyrine/Phenazone	CC1=CC(=O)N(N1C)C2=CC=CC=C2	8.64

Atenolol	CC(C)NCC(COC1=CC=C(C=C1)CC(=O)N)O	3.78
Azinophos-ethyl	CCOP(=S)(OCC)SCN1C(=O)C2=CC=CC=C2N=N1	19.19
Bentazon	CC(C)N1C(=O)C2=CC=CC=C2NS1(=O)=O	14.47
Bezafibrate	CC(C)(C(=O)O)OC1=CC=C(C=C1)CCNC(=O)C2=CC=C(C=C2)Cl	15.92
Caffeine	CN1C=NC2=C1C(=O)N(C(=O)N2C)C	6.83
Carbamazepine	C1=CC=C2C(=C1)C=CC3=CC=CC=C3N2C(=O)N	13.23
Climbazole	CC(C)(C)C(=O)C(N1C=CN=C1)OC2=CC=C(C=C2)Cl	14.3
Clindamycin	CCCC1CC(N(C1)C)C(=O)NC(C2C(C(C(C(O2)SC)O)O)O)C(C)Cl	10.62
Cyanazine	CCNC1=NC(=NC(=N1)Cl)NC(C)(C)C#N	12.98
DEET	CCN(CC)C(=O)C1=CC=CC(=C1)C	14.81
Desmetryne	CC(C)NC1=NC(=NC(=N1)NC)SC	11.1
Diclofenac	C1=CC=C(C(=C1)CC(=O)O)NC2=C(C=CC=C2Cl)Cl	18.31
Diglyme/ diethylene glycol dimethyl	COCCOCCOC	5.68
Dimefuron	CC(C)(C)C1=NN(C(=O)O1)C2=C(C=C(C=C2)NC(=O)N(C)C)Cl	16.08
Dimethanamid-p	CC1=CSC(=C1N(C(C)COC)C(=O)CCl)C	17.33
Diuron	CN(C)C(=O)NC1=CC(=C(C=C1)Cl)Cl	15.06
Ethofumesate	CCOC1C(C2=C(O1)C=CC(=C2)OS(=O)(=O)C)(C)C	18.46
Fenuron	CN(C)C(=O)NC1=CC=CC=C1	9.43
Fipronil	C1=C(C=C(C(=C1Cl)N2C(=C(C(=N2)C#N)S(=O)C(F)(F)F)N)Cl)C(F)(F)F	19.89
Fluroxypyr	C(C(=O)O)OC1=NC(=C(C(=C1Cl)N)Cl)F	12.84
Furosemide	C1=COC(=C1)CNC2=CC(=C(C=C2C(=O)O)S(=O)(=O)N)Cl	13.42
Gemfibrozil	CC1=CC(=C(C=C1)C)OCCCC(C)(C)C(=O)O	20.12
Ibuprofen	CC(C)CC1=CC=C(C=C1)C(C)C(=O)O	18.73
Lincomycin	CCCC1CC(N(C1)C)C(=O)NC(C2C(C(C(C(O2)SC)O)O)O)C(C)O	6.85
Linuron	CN(C(=O)NC1=CC(=C(C=C1)Cl)Cl)OC	17.22
MCPA	CC1=C(C=CC(=C1)Cl)OCC(=O)O	15.31
MCPP-p	CC1=C(C=CC(=C1)Cl)OC(C)C(=O)OCC2=C(C(=C(C(=C2F)F)F)F)F	16.49
Metamitron	CC1=NN=C(C(=O)N1N)C2=CC=CC=C2	
Methoxyfenozide	CC1=CC(=CC(=C1)C(=O)N(C(C)(C)C)NC(=O)C2=C(C(=CC=C2)OC)C)C	18.37
Metoprolol	CC(C)NCC(COC1=CC=C(C=C1)CCOC)O	9.4
N-Phenyl urea	C1=CC=C(C=C1)NC(=O)N	7.69
Pentachlorophenol	C1(=C(C(=C(C(=C1Cl)Cl)Cl)Cl)Cl)O	20.7
PFBA	C(=O)(C(C(C(F)(F)F)(F)F)(F)F)O	8.93
PFOA	C(=O)(C(C(C(C(C(C(C(F)(F)F)(F)F)(F)F)(F)F)(F)F)(F)F)(F)F)O	16.75
Prometon	CC(C)NC1=NC(=NC(=N1)OC)NC(C)C	11.55
Prometryne	CC(C)NC1=NC(=NC(=N1)SC)NC(C)C	14.52
Propazine	CC(C)NC1=NC(=NC(=N1)Cl)NC(C)C	16.36
Prosulfocarb	CCCN(CCC)C(=O)SCC1=CC=CC=C1	22.01
Saccharin	C1=CC=C2C(=C1)C(=O)NS2(=O)=O	5.31
Simetryne	CCNC1=NC(=NC(=N1)SC)NCC	11.1
Sotalol	CC(C)NCC(C1=CC=C(C=C1)NS(=O)(=O)C)O	4.57
Spiromesifen	CC1=CC(=C(C(=C1)C)C2=C(C3(CCCC3)OC2=O)OC(=O)CC(C)(C)C)C	
Sulfamethazine	CC1=CC(=NC(=N1)NS(=O)(=O)C2=CC=C(C=C2)N)C	8.37

Sulfamethoxazole	CC1=CC(=NO1)NS(=O)(=O)C2=CC=C(C=C2)N	10.73
Tebuthiuron	CC(C)(C)C1=NN=C(S1)N(C)C(=O)NC	
Terbumeton	CCNC1=NC(=NC(=N1)OC)NC(C)(C)C	11.78
Tramadol	CN(C)CC1CCCCC1(C2=CC(=CC=C2)OC)O	9.43
Triclosan	C1=CC(=C(C=C1Cl)O)OC2=C(C=C(C=C2)Cl)Cl	21.11
Triethyl phosphate	CCOP(=O)(OCC)OCC	10.94
Trimethoprim	COC1=CC(=CC(=C1OC)OC)CC2=CN=C(N=C2N)N	7.82
Venlafaxine	CN(C)CC(C1=CC=C(C=C1)OC)C2(CCCCC2)O	10.96

III. Suspect list for parent compounds

Table S3. OMPs previously detected in the Haaksbergen field and used as parent compounds list, with type of chemical, SMILES, and retention times (RT) in the LC column in minutes. The RT was not available for OMPs not present in the performance standard list (Table S2).

**Name**	**Type**	**SMILES**	**RT (min)**
1H-benzotriazole	Industrial chemical	C1=CC2=NNN=C2C=C1	7.96
1-Hydroxyibuprofen	Pharmaceutical	CC(C)C(C1=CC=C(C=C1)C(C)C(=O)O)O	
2-(Methylamino)pyridine	Pharmaceutical	CNC1=CC=CC=N1	
2,4,5-Trichlorophenoxyacetic acid	Pesticide	C1=C(C(=CC(=C1Cl)Cl)Cl)OCC(=O)O	
2,4-Dichlorophenoxyacetic acid	Pesticide	C1=CC(=C(C=C1Cl)Cl)OCC(=O)O	15.26
2,6-Dichlorobenzamide	Pesticide	C1=CC(=C(C(=C1)Cl)C(=O)N)Cl	8.18
2-Hydroxyquinoline	Industrial chemical	C1=CC=C2C(=C1)C=CC(=O)N2	9.16
3-Hydroxycarbamazepine	Pharmaceutical	C1=CC=C2C(=C1)C=CC3=C(N2C(=O)N)C=C(C=C3)O	11.64
4,6-Dinitro-o-cresol	Pesticide	CC1=CC(=CC(=C1O)[N+](=O)[O-])[N+](=O)[O-]	15.91
4-Hydroxyquinoline	Industrial chemical	C1=CC=C2C(=C1)C(=O)C=CN2	5.98
6-hydroxyquinoline	Industrial chemical	C1=CC2=C(C=CC(=C2)O)N=C1	
Acesulfame K	Industrial chemical	CC1=CC(=O)[N-]S(=O)(=O)O1.[K+]	3.19
Paracetamol	Pharmaceutical	CC(=O)NC1=CC=C(C=C1)O	
Ametryn	Pesticide	CCNC1=NC(=NC(=N1)SC)NC(C)C	12.82
Antipyrine	Pharmaceutical	CC1=CC(=O)N(N1C)C2=CC=CC=C2	8.64
Atenolol	Pharmaceutical	CC(C)NCC(COC1=CC=C(C=C1)CC(=O)N)O	3.78
Atrazine	Pesticide	CCNC1=NC(=NC(=N1)Cl)NC(C)C	
Azinophos-ethyl	Pesticide	CCOP(=S)(OCC)SCN1C(=O)C2=CC=CC=C2N=N1	19.19
Bentazon	Pesticide	CC(C)N1C(=O)C2=CC=CC=C2NS1(=O)=O	14.47
Bezafibrate	Pharmaceutical	CC(C)(C(=O)O)OC1=CC=C(C=C1)CCNC(=O)C2=CC=C(C=C2)Cl	15.92
Caffeine	Pharmaceutical	CN1C=NC2=C1C(=O)N(C(=O)N2C)C	6.83
Carbamazepine	Pharmaceutical	C1=CC=C2C(=C1)C=CC3=CC=CC=C3N2C(=O)N	13.23
Chloroxuron	Pesticide	CN(C)C(=O)NC1=CC=C(C=C1)OC2=CC=C(C=C2)Cl	
Climbazole	Pharmaceutical	CC(C)(C)C(=O)C(N1C=CN=C1)OC2=CC=C(C=C2)Cl	14.3
Clindamycin	Pharmaceutical	CCCC1CC(N(C1)C)C(=O)NC(C2C(C(C(C(O2)SC)O)O)O)C(C)Cl	10.62
Clofibric acid	Pharmaceutical	CC(C)(C(=O)O)OC1=CC=C(C=C1)Cl	
Clomazone	Pesticide	CC1(CON(C1=O)CC2=CC=CC=C2Cl)C	
Cyanazine	Pesticide	CCNC1=NC(=NC(=N1)Cl)NC(C)(C)C#N	12.98

DEET	Pesticide	CCN(CC)C(=O)C1=CC=CC(=C1)C	14.81
Desmetryne	Pesticide	CC(C)NC1=NC(=NC(=N1)NC)SC	11.1
Diclofenac	Pharmaceutical	C1=CC=C(C(=C1)CC(=O)O)NC2=C(C=CC=C2Cl)Cl	18.31
Diglyme	Industrial chemical	COCCOCCOC	5.68
Dimefuron	Pesticide	CC(C)(C)C1=NN(C(=O)O1)C2=C(C=C(C=C2)NC(=O)N(C)C)Cl	16.08
Dimethametryn	Pesticide	CCNC1=NC(=NC(=N1)SC)NC(C)C(C)C	
Dimethanamid-p	Pharmaceutical	CC1=CSC(=C1N(C(C)COC)C(=O)CCl)C	17.33
Diuron	Pesticide	CN(C)C(=O)NC1=CC(=C(C=C1)Cl)Cl	15.06
Ethofumesate	Pesticide	CCOC1C(C2=C(O1)C=CC(=C2)OS(=O)(=O)C)(C)C	18.46
Fenuron	Pesticide	CN(C)C(=O)NC1=CC=CC=C1	9.43
Fipronil	Pesticide	C1=C(C=C(C(=C1Cl)N2C(=C(C(=N2)C#N)S(=O)C(F)(F)F)N)Cl)C(F)(F)F	19.89
Fluometuron	Pesticide	CN(C)C(=O)NC1=CC=CC(=C1)C(F)(F)F	
Fluoxastrobin (, trans-)	Pesticide	CO\N=C(\C1=NOCCO1)C1=C(OC2=C(F)C(OC3=C(Cl)C=CC=C3)=NC=N2)C=CC=C1	
Fluroxypyr	Pesticide	C(C(=O)O)OC1=NC(=C(C(=C1Cl)N)Cl)F	12.84
Furosemide	Pharmaceutical	C1=COC(=C1)CNC2=CC(=C(C=C2C(=O)O)S(=O)(=O)N)Cl	13.42
Gemfibrozil	Pharmaceutical	CC1=CC(=C(C=C1)C)OCCCC(C)(C)C(=O)O	20.12
HFPO-DA	Industrial chemical	C(=O)(C(C(F)(F)F)(OC(C(C(F)(F)F)(F)F)(F)F)F)O	
Ibuprofen	Pharmaceutical	CC(C)CC1=CC=C(C=C1)C(C)C(=O)O	18.73
Imidacloprid	Pharmaceutical	C1CN(C(=N[N+](=O)[O-])N1)CC2=CN=C(C=C2)Cl	
Ioxynil	Pesticide	C1=C(C=C(C(=C1I)O)I)C#N	
Ketoprofen	Pharmaceutical	CC(C1=CC(=CC=C1)C(=O)C2=CC=CC=C2)C(=O)O	
Lincomycin	Pharmaceutical	CCCC1CC(N(C1)C)C(=O)NC(C2C(C(C(C(O2)SC)O)O)O)C(C)O	6.85
Linuron	Pesticide	CN(C(=O)NC1=CC(=C(C=C1)Cl)Cl)OC	17.22
MCPA	Pesticide	CC1=C(C=CC(=C1)Cl)OCC(=O)O	15.31
MCPB	Pesticide	CC1=C(C=CC(=C1)Cl)OCCCC(=O)O	
MCPP	Pesticide	CC1=C(C=CC(=C1)Cl)OC(C)C(=O)OCC2=C(C(=C(C(=C2F)F)F)F)F	16.49
Mesotrione	Pesticide	CS(=O)(=O)C1=CC(=C(C=C1)C(=O)C2C(=O)CCCC2=O)[N+](=O)[O-]	
Methabenzthiazuron	Pesticide	CNC(=O)N(C)C1=NC2=CC=CC=C2S1	
Methoxyfenozide	Pesticide	CC1=CC(=CC(=C1)C(=O)N(C(C)(C)C)NC(=O)C2=C(C(=CC=C2)OC)C)C	18.37
Metolachlor-s	Pesticide	CCC1=CC=CC(=C1N(C(C)COC)C(=O)CCl)C	
Metoprolol	Pharmaceutical	CC(C)NCC(COC1=CC=C(C=C1)CCOC)O	9.4
Metribuzin	Pesticide	CC(C)(C)C1=NN=C(N(C1=O)N)SC	
Naproxen	Pharmaceutical	CC(C1=CC2=C(C=C1)C=C(C=C2)OC)C(=O)O	
Neburon	Pesticide	CCCCN(C)C(=O)NC1=CC(=C(C=C1)Cl)Cl	
Nicosulfuron	Pesticide	CN(C)C(=O)C1=C(N=CC=C1)S(=O)(=O)NC(=O)NC2=NC(=CC(=N2)OC)OC	
N-Phenyl urea	Industrial chemical	C1=CC=C(C=C1)NC(=O)N	7.69
Pentachlorophenol	Pharmaceutical	C1(=C(C(=C(C(=C1Cl)Cl)Cl)Cl)Cl)O	20.7
PFBA	Industrial chemical	C(=O)(C(C(C(F)(F)F)(F)F)(F)F)O	8.93
PFOA	Industrial chemical	C(=O)(C(C(C(C(C(C(C(F)(F)F)(F)F)(F)F)(F)F)(F)F)(F)F)(F)F)O	16.75
Prometon	Pesticide	CC(C)NC1=NC(=NC(=N1)OC)NC(C)C	11.55

Prometryne	Pesticide	CC(C)NC1=NC(=NC(=N1)SC)NC(C)C	14.52
Propanil	Pesticide	CCC(=O)NC1=CC(=C(C=C1)Cl)Cl	
Propazine	Pesticide	CC(C)NC1=NC(=NC(=N1)Cl)NC(C)C	16.36
Propoxur	Pesticide	CC(C)OC1=CC=CC=C1OC(=O)NC	
Prosulfocarb	Pesticide	CCCN(CCC)C(=O)SCC1=CC=CC=C1	22.01
Pyridafol	Industrial chemical	C1=CC=C(C=C1)C2=NNC(=CC2=O)Cl	
Saccharin	Industrial chemical	C1=CC=C2C(=C1)C(=O)NS2(=O)=O	5.31
Simetryne	Pesticide	CCNC1=NC(=NC(=N1)SC)NCC	11.1
Sotalol	Pharmaceutical	CC(C)NCC(C1=CC=C(C=C1)NS(=O)(=O)C)O	4.57
Sulfamethazine	Pharmaceutical	CC1=CC(=NC(=N1)NS(=O)(=O)C2=CC=C(C=C2)N)C	8.37
Sulfamethoxazole	Pharmaceutical	CC1=CC(=NO1)NS(=O)(=O)C2=CC=C(C=C2)N	10.73
Teflubenzuron	Pesticide	C1=CC(=C(C(=C1)F)C(=O)NC(=O)NC2=CC(=C(C(=C2F)Cl)F)Cl)F	
Terbacil	Pesticide	CC1=C(C(=O)N(C(=O)N1)C(C)(C)C)Cl	
Terbumeton	Pesticide	CCNC1=NC(=NC(=N1)OC)NC(C)(C)C	11.78
Thiacloprid	Pesticide	C1CSC(=NC#N)N1CC2=CN=C(C=C2)Cl	
Tramadol	Pharmaceutical	CN(C)CC1CCCCC1(C2=CC(=CC=C2)OC)O	9.43
Triazophos	Pesticide	CCOP(=S)(OCC)OC1=NN(C=N1)C2=CC=CC=C2	
Triclosan	Industrial chemical	C1=CC(=C(C=C1Cl)O)OC2=C(C=C(C=C2)Cl)Cl	21.11
Triethyl phosphate	Industrial chemical	CCOP(=O)(OCC)OCC	10.94
Trimethoprim	Pharmaceutical	COC1=CC(=CC(=C1OC)OC)CC2=CN=C(N=C2N)N	7.82
Venlafaxine	Pharmaceutical	CN(C)CC(C1=CC=C(C=C1)OC)C2(CCCCC2)O	10.96

IV. patRoon identification level rules

Table S4. Rules for the assignation of the identification levels in patRoon. Level 3b was not applicable in this study, as the suspect lists did not contain fragment information. (Adapted from the handbook of patRoon[4] updated on 24/04/2024).

Level	Description	Rules
1	Target match	Retention time deviates <12 seconds from the suspect list. In our research, level 1 was assigned to the parent compounds detected in both the performance standard and the samples with NTA.
2a	Good MS/MS library match	Suspect is top-ranked in the compound annotation results.<br>The individualMoNAScore (MetFrag) is at least 0.9 and no other candidates were matched with the MS library.
3a	Fair library match	The individualMoNAScore is at least 0.4.
3b	Known MS/MS match	At least 3 (or all if the suspect list contains less) fragments from the suspect list must match.
3c	Good in-silico MS/MS match	The annotation MS/MS similarity (annSimComp column) is at least 0.7.
4a	Good formula MS/MS match	Suspect is top ranked formula candidate, annotation MS/MS similarity (annSimForm column) is at least 0.7 and isotopic match (isoScore) of at least 0.5. The latter two scores are at least 0.2 higher than next best ranked candidate.
4b	Good formula isotopic pattern match	Suspect is top ranked formula candidate and isotopic match (isoScore) of at least 0.9 and at least 0.2 higher than next best ranked candidate.
5	Unknown	All else.

References

1. Narain-Ford, D. M., van Wezel, A. P., Helmus, R., Dekker, S. C. & Bartholomeus, R. P. Soil self-cleaning capacity: Removal of organic compounds during sub-surface irrigation with sewage effluent. *Water Res.* **226**, 119303 (2022).

2. Been, F. *et al.* Risk-based prioritization of suspects detected in riverine water using complementary chromatographic techniques. *Water Res.* **204**, 117612 (2021).

3. Brunner, A. M. *et al.* Monitoring transformation product formation in the drinking water treatments rapid sand filtration and ozonation. *Chemosphere* **214**, 801–811 (2019).

4. Helmus, R. *patRoon Handbook*.
